# Comparing the Performance of Popular MEG/EEG Artifact Correction Methods in an Evoked-Response Study

**DOI:** 10.1155/2016/7489108

**Published:** 2016-07-21

**Authors:** Niels Trusbak Haumann, Lauri Parkkonen, Marina Kliuchko, Peter Vuust, Elvira Brattico

**Affiliations:** ^1^Center for Music in the Brain, Department of Clinical Medicine, Aarhus University and Royal Academy of Music, Aarhus/Aalborg, Nørrebrogade 44, Building 10G, 4th and 5th floor, 8000 Aarhus C, Denmark; ^2^Department of Neuroscience and Biomedical Engineering, Aalto University School of Science, P.O. Box 12200, 00076 Aalto, Finland; ^3^Cognitive Brain Research Unit, Institute of Behavioural Sciences, University of Helsinki, P.O. Box 9 (Siltavuorenpenger 1 B), 00014 Helsinki, Finland; ^4^BioMag Laboratory, HUS Medical Imaging Center, University of Helsinki and Helsinki University Hospital, P.O. Box 340, Hospital District of Helsinki and Uusimaa, 00029 Helsinki, Finland

## Abstract

We here compared results achieved by applying popular methods for reducing artifacts in magnetoencephalography (MEG) and electroencephalography (EEG) recordings of the auditory evoked Mismatch Negativity (MMN) responses in healthy adult subjects. We compared the Signal Space Separation (SSS) and temporal SSS (tSSS) methods for reducing noise from external and nearby sources. Our results showed that tSSS reduces the interference level more reliably than plain SSS, particularly for MEG gradiometers, also for healthy subjects not wearing strongly interfering magnetic material. Therefore, tSSS is recommended over SSS. Furthermore, we found that better artifact correction is achieved by applying Independent Component Analysis (ICA) in comparison to Signal Space Projection (SSP). Although SSP reduces the baseline noise level more than ICA, SSP also significantly reduces the signal—slightly more than it reduces the artifacts interfering with the signal. However, ICA also adds noise, or correction errors, to the waveform when the signal-to-noise ratio (SNR) in the original data is relatively low—in particular to EEG and to MEG magnetometer data. In conclusion, ICA is recommended over SSP, but one should be careful when applying ICA to reduce artifacts on neurophysiological data with relatively low SNR.

## 1. Introduction

Recordings of evoked-responses (also known as event-related potentials, ERPs, or event-related fields, ERFs) with electroencephalography (EEG) or magnetoencephalography (MEG) are widely used methods in cognitive and clinical neuroscience. One of the major challenges in research and clinical applications of evoked-responses is the prevalent strongly interfering electromagnetic signals from external objects and devices in the surrounding MEG or EEG measurement environment as well as nearby mechanical and biological electromagnetic sources originating from the head and other parts of the body of the subject. Since the interfering environmental noise from, for example, laboratory mechanics and electronic devices may be several orders of magnitude stronger than the brain signals of interest (for a review, see, e.g., [1]), it is necessary to remove this noise from the recordings during or after the measurements. Moreover, nonencephalic electromagnetic activity, such as that from the eyes and from the cardiac and facial muscles, is also recorded by EEG or MEG and can be up to a thousand times stronger than the encephalic signal of interest [1]. Since some of these interfering artifactual signals can be synchronous with the brain signal of interest, significant parts of the continuous measurement can be contaminated by artifacts. Hence, to ensure a reliable measurement, it is necessary, in addition to applying an average measure of an evoked-response across multiple time-locked data segments, also to omit or correct the data contaminated with artifacts.

In the clinical routine, data from patients having a limited control of muscular activity (such as stroke or dementia patients or preterm infants) or with ferromagnetic implants (such as cochlear implantees) typically contain a considerable amount of artifacts. The time constraints of experiments and tests on clinical populations exclude the possibility of a large number of trials that would allow discarding the artefactual ones. A viable alternative to simply rejecting parts of the recorded data is that of correcting the data. Both in clinical patient recordings and in experimental settings with healthy subjects, strong electromagnetic noise from electronic devices, static electricity, and in particular with regard to EEG also the 50/60-Hz power-line noise may interfere significantly with the measurements [2]. When recording EEG in conjunction with transcranial magnetic stimulation (TMS), methods have been developed for reducing the strong TMS artifacts appearing in the recording [3]. In other cases where two neuroimaging modalities are employed simultaneously, special care must also be taken to reduce artifacts originating from the other modality. For recording EEG concurrently with functional magnetic resonance imaging (fMRI), it is necessary to reduce both imaging artifacts caused by the switching gradient fields [4] and ballistocardiogram artifacts caused by the subjects heart beats moving the skin and electrodes in relation to the strong magnetic field within the MRI scanner [5]. Furthermore, in combined EEG/MEG recordings one should be aware that eddy currents in the electrodes induce magnetic fields, which may introduce artifacts in the MEG recordings for signals in higher frequency ranges; however, signals at frequencies below 100 Hz are not critically affected by these types of artifacts [6].

Apart from external artifact sources, it is important to reduce the influence of the internal artifacts originating from the head and the rest of the body of the subject. Typically, MEG and EEG recordings are contaminated by relatively strong artifacts caused by the eyes [2, 7–9]. They can either be eye blinks (picked up mostly by the vertical EOG) contaminating particularly the lower frequencies or be saccades (visible mostly in horizontal EOG) also interfering at higher frequency ranges, where certain saccadic spike artifacts resemble high-frequency muscular artifacts [9]. Another typically interfering internal artifact is due to the electric activity of the cardiac muscle, measured by electrocardiography (ECG or EKG) [2, 10]. Also, noises from different types of muscular activity, seen in electromyographic (EMG) signals, are also a typical issue in MEG and EEG recordings [2, 11]. These muscular artifacts may be caused by mastication (chewing), deglutition (tongue movement), and respiration [2].

Different methods have been developed to reduce the influence of externally and internally originating artifacts. The externally originating interference can be minimized by applying physical shielding techniques in the laboratory [12], by using gradiometer sensors instead of magnetometers, by subtracting measurements of the external noise signals recorded by one or more reference sensors or by applying online or offline spatial filtering methods. MEG systems by Elekta Oy (Helsinki, Finland) comprise both magnetometers and gradiometers and they employ spatial filtering techniques such as Signal Space Projection (SSP) and Signal Space Separation (SSS) and its temporal extension (tSSS) implemented in the Elekta Neuromag® MaxFilter*™* software [13]. The SSS method is based on Maxwell equations and the multichannel measurement of the magnetic field distribution; by using a basis comprising spherical harmonic functions, contributions of signal sources within the sensor array (brain signals) can be separated from sources external to the array [13–18]. Since SSS is purely a spatial filter, which only reduces noise originating from the external sources, it retains also those brain signals that oscillate at the same frequency as an external noise source [14]. However, nearby sources of artifacts caused by, for example, movement of magnetic materials, such as dental braces or implants, cannot be sufficiently reduced by applying SSS alone.

The tSSS method is additionally able to filter out interferences from artifactual sources in the space between the brain and the MEG sensor array, by reducing signals in the common subspace through comparisons of the time series in the internal and external spaces. For instance, it has been shown that tSSS makes it possible to locate brain sources on the cortex with beamformer methods in clinical patients, although these patients wore strongly magnetically interfering dental braces; thereby, tSSS seems to allow extending the clinical population compatible with MEG [17].

The internally originating artifacts can be reduced by applying band-pass filtering [19] and component analysis such as Principal Component Analysis (PCA) or Independent Component Analysis (ICA) [20–43] or by recording the artifacts to be removed, identifying their contribution to the data by means of linear regression and subtracting them out [10, 44, 45]. Also, methods for ignoring the artifactual sources have been implemented as part of source analysis algorithms [46, 47]. With regard to the component analysis approaches, PCA is applied to estimate components explaining the highest variance in the data, such as strong artifacts. ICA is able to estimate components that explain variance originating from statistically independent sources, thereby reducing the risk of including signals of interest in the derived artifact components. The ICA algorithms, and, in particular, the infomax version of ICA, have gained popularity as an efficient method for separating the recorded signals into statistically independent components [43]. By inspecting the independent components, only the artifactual components can be rejected to reduce the influence of the artifacts on the data. An alternative method of Signal Space Projection (SSP) has gained some popularity in open source software packages [48, 49]. SSP also decomposes the data into components, often based on a prior PCA; however, in contrast with ICA, these components may not be statistically independent, and therefore there is a risk that artifactual and brain signals of interest may be reduced simultaneously [48].

To investigate the performances and risks of using different popular artifact correction methods, we compared here results achieved by applying SSS, tSSS, ICA, and SSP. We chose to study the performance of the correction methods on the Mismatch Negativity (MMN) response, which is a well-known evoked-response [50, 51]. In particular, we wanted to investigate (1) whether tSSS improves the data quality in healthy subjects not wearing any magnetically disturbing implants; (2) whether the faster SSS alternative performs as well as the more computationally demanding tSSS; and (3) whether ICA is preferable over SSP (or vice versa) for reduction of typical artifacts in healthy subjects.

## 2. Methods

### 2.1. Participants

A sample of ten volunteers from a larger database named “Tunteet” was chosen (for a description of the experimental protocol, see Kliuchko et al., submitted). The participants were six females and four males. Three participants were nonmusicians, three were amateur musicians, and four were professional musicians. All participants were right-handed, and their average age was 24.8 years (range 18–35 years). Written informed consent was obtained from each participant, and the study was approved by the local ethics committee.

### 2.2. Experimental Paradigm

The participants listened to a melody pattern of 2100 ms, repeated with variations during ~25 minutes. The melody patterns were created from digital piano tones (McGill University Master Samples) and followed the rules of Western tonal music. All melodies started with a triad (300 ms) followed by four single tones (two of 125 ms and two of 300 ms) and an ending tone (575 ms) all separated by 50 ms silent gap. Between all melodies there was a silent gap of 125 ms. Deviations of six types were inserted into the melody patterns to evoke MMN responses to a deviant tone as compared with corresponding unaltered standard. The deviants are explained in Table 1 [52–54].

In total, the tested sample contained 120 brain responses, which comprised the responses to the six standard and six deviant conditions from each of the ten participants.

### 2.3. Data Acquisition

The simultaneous MEG and EEG data were collected at the BioMag Laboratory of the Helsinki University Central Hospital. The measurements were performed in an electrically and magnetically shielded room (ETS-Lindgren Euroshield, Eura, Finland) with Vectorview*™* 306-channel MEG system (Elekta Neuromag, Elekta Oy, Helsinki, Finland) equipped with a compatible EEG system. The MEG system had 102 triple-sensor elements, each comprising two orthogonal planar gradiometers and one magnetometer. A 64-channel EEG electrode cap was used. The reference electrode was placed on the nose tip and the ground electrode was on the right cheek. Blinks, as well as vertical and horizontal eye movements, were measured with four electrodes attached above and below the left eye and close to the external eye corners on both sides. Four head position indicator coils were placed on top of the EEG cap. Their positions were determined with respect to the nasion and preauricular points by an Isotrak 3D digitizer (Polhemus, Colchester, VT, USA). MEG and EEG data were recorded with a sample rate of 600 Hz.

During the measurement, subjects were comfortably seated and watched a silenced movie with subtitles. The stimuli were presented with Presentation software (Neurobehavioral Systems, Ltd.). The sound was delivered through a pair of pneumatic headphones at individually adjusted loudness.

### 2.4. Artifact Correction

 Elekta Neuromag MaxFilter 2.2 Signal Space Separation (SSS) and temporal Signal Space Separation (tSSS) [13, 16] were applied separately to compare their individual performance. For both SSS and tSSS, we used the default inside expansion order of 8, outside expansion order of 3, automatic optimization of both inside and outside bases, and automatic detection and correction of bad MEG channels. Additionally, for both SSS and tSSS the specific fine calibration and cross talk correction data for the recording site and date were applied. For the tSSS, we used the default subspace correlation limit of 0.980 and raw data buffer length of 10 seconds. The spatially filtered data were saved in 32-bit float format at a sampling rate of 600 Hz.

Correction for internal artifacts with Signal Space Projection (SSP) was performed with the MNE Python version 0.11.0 released with the MNE software version 2.7.4-3485 [55, 56]. We applied the default automatic settings, where two principal components per artifact type are detected for eye artifacts and for cardiac artifacts. Subsequently, the detected ocular and cardiac artifact component projections were removed from the data.

Independent Component Analysis- (ICA-) based correction for internal artifacts was achieved by applying the logistic infomax algorithm implemented in the* runica* function [57] for MATLAB® (MathWorks, Natick, Massachusetts). First, the data were reduced to 64 principal components. The independent components were then estimated for the EEG channels, MEG magnetometers, and MEG planar gradiometers separately. The resulting components were inspected, and one component projection per vertical eye movement, horizontal eye movement, or cardiac artifact type (explaining most variance) was removed from the data, when the artifact component was observed. On average, the total number of observed artifact components per subject was 1.7 (1-2) for the EEG, 1.8 (1–3) for the MEG magnetometers, and 2.0 (1–3) for the MEG gradiometers.

### 2.5. Data Analysis

Event-related EEG and MEG responses were extracted as single-trial epochs with a time window of 0 to 400 ms after the standard or deviant tone onset. The trials were baseline-corrected by applying a baseline of −100 to 0 ms before the tone onset. Since the planar gradiometer sensors measure along two orthogonal directions, the data from each pair of longitudinal and latitudinal sensors were combined by applying the Pythagorean distance formula, as implemented in the FieldTrip toolbox for MATLAB (Donders Institute for Brain, Cognition and Behaviour/Max Planck Institute, Nijmegen, Netherlands) [58]; $d=\sqrt{{longitudinal}^{\text{2}}+{latitudinal}^{\text{2}}}$.

**Figure 1:**
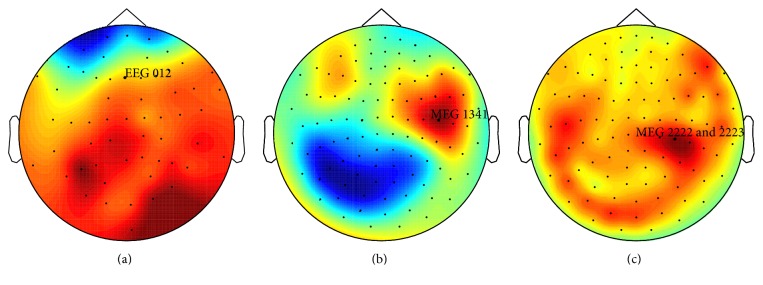
Topographical plots of MMN responses (without correction for internal artifacts). Showing topographical plots of MMN responses to the mistuning deviants, which elicited the strongest and most consistent MMN responses in the tested sample in the EEG (a), MEG magnetometers (b), and MEG gradiometers (c). The plots are based on grand averages from uncorrected data (only preprocessed with tSSS) by applying a time window from 125 to 155 ms after the tone onset.

For the sake of clarity, we performed the subsequent analyses on one channel of each sensor type, those in which the highest MMN amplitude was measured within a typical MMN latency range of 75–200 ms. In this case, we analyzed the event-related waveforms from EEG channel 012 (frontal site), magnetometer channel MEG 1341 (right temporal site), and the combined planar gradiometer channels MEG 2222 and 2223 (right temporal site) (see Figure 1). These analyzed channels behaved reliably and were not detected as bad channels or subjected to any additional correction.

We measured the MMN amplitude in response to each type of deviant tone by taking the average value across the time window from 125 to 155 ms after the tone onset. To compare the noise levels after utilizing each artifact correction method, we first used a baseline standard deviation measure. Since a flat baseline is desirable, we applied a baseline standard deviation (STD) measure to show the flatness of the baseline in a single trial (where lower baseline STD means a more flat baseline) [59]. We calculated the standard deviation across the baseline time points from −100 to 0 ms (in relation to the stimulus onset) in each trial separately and extracted the mean baseline STD across trials. Also, minimal variance in the measured signal across trials is desirable. Therefore, we also calculated the signal STD across trials for each time point in 125 to 155 ms:

(1) $$\begin{array}{c} \sigma_{\overset{-}{x}}=\sqrt{\frac{\sum_{i=1}^{n}{\left(x_{i}-\overset{-}{x}\right)}^{2}}{N}}, \end{array}$$

where *x* is the measured value, $\overset{-}{x}$ is the mean value, *i* is the trial number, and *N* is the total number of trials [18] and we averaged these values to obtain the mean signal STD. For additional comparisons, we applied a signal-to-noise ratio measure; $SNR=amplitude/\sigma_{\overset{-}{x}}$ [18].

The Mismatch Negativity (MMN) evoked-response is analyzed by comparing the average response to the deviant stimulus with the average response to the standard stimulus [50, 51]. Also, MMN waveforms are conventionally calculated by subtracting the average response to the standard stimulus from the average response to the deviant stimulus [50, 51]. However, we here analyze the noise levels across multiple single-trial MMN responses, which does not allow us to create difference waveforms by simply subtracting particular pairs of deviant and standard trials among multiple equally possible pairings of trials. The responses to both deviant and standard stimuli are relevant for any subsequent analyses of MMN, and it is therefore important to know the noise levels of responses to both stimuli. Therefore, we here analyzed the noise levels in the responses to both the deviant and standard stimuli.

Statistical comparisons were made with SPSS version 20 (IBM, Armonk, New York, USA). Since the resulting values were not normally distributed, we applied the Wilcoxon signed-rank test to compare the values achieved after utilizing the different artifact correction methods.

## 3. Results

### 3.1. SSS and tSSS

**Figure 2:**
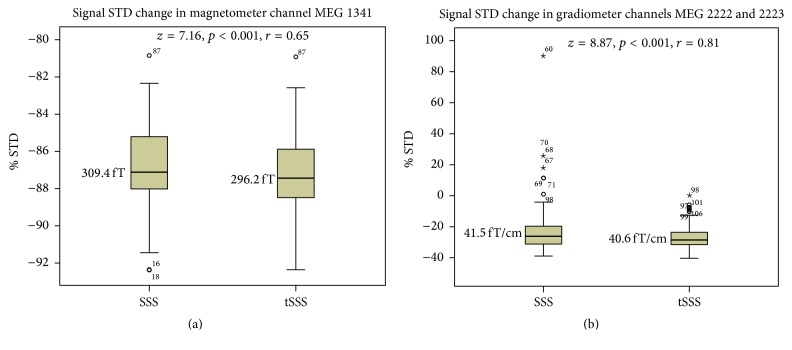
Percentagewise standard deviation (STD) reduction in the signal latency range achieved by applying the SSS or tSSS methods in comparison to the level (at 0% STD) in the raw data recording without spatial filtering. The differences are shown for the amplitude peak channel of the magnetometers (a) and gradiometers (b) in Tukey box plots. Bold horizontal lines indicate medians, brown boxes indicate the first 50 percent of the case range, and vertical lines indicate cases within the 1.5 interquartile range from the edge of the 50% of the cases. Outliers more than 1.5 (circles) or 3.0 (stars) interquartile ranges from the edge of the 50% of the cases are denoted with circles or stars, and case numbers are provided for each outlier. The median values are shown in femtoteslas (fT) and femtoteslas per centimeter (fT/cm). *z* and *r* indicate the effect size, and *p* indicates the level of statistical significance.

A statistically significant and slightly better reduction of the signal standard deviation (STD) is achieved by applying tSSS in comparison to SSS for for both the MEG magnetometer and gradiometer data (see Figure 2). Importantly, in 6% (7/120) of the tested cases, the signal STD actually increases when applying SSS to the MEG gradiometer data, whereas the signal STD either is retained or decreases when the tSSS method is applied.

### 3.2. ICA and SSP

**Figure 3:**
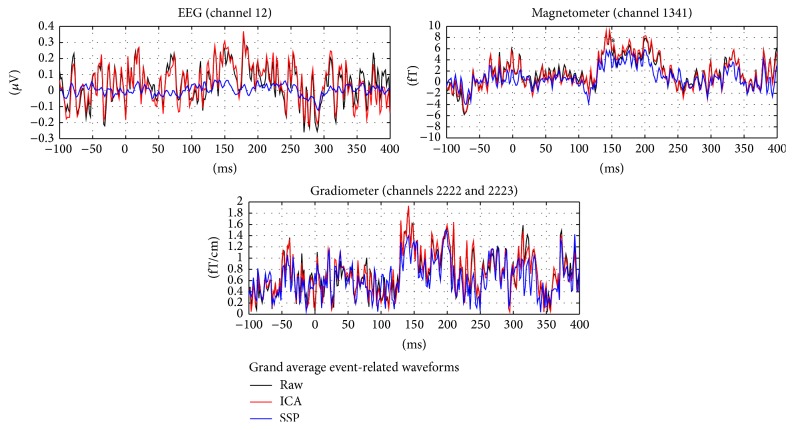
Grand average event-related waveforms. Showing the channels with the signal peak amplitude among the EEG channels, magnetometers, and gradiometers, without any artifact correction (black) and after artifact correction based on Independent Component Analysis (ICA) (red) and Signal Space Projection (SSP) (blue).

From grand average waveforms of the event-related responses (across all participants and conditions), it can be seen that the SSP-based artifact correction reduces the signal amplitude, whereas the signal amplitude is similar before and after the artifact correction based on ICA (see Figure 3).

**Figure 4:**
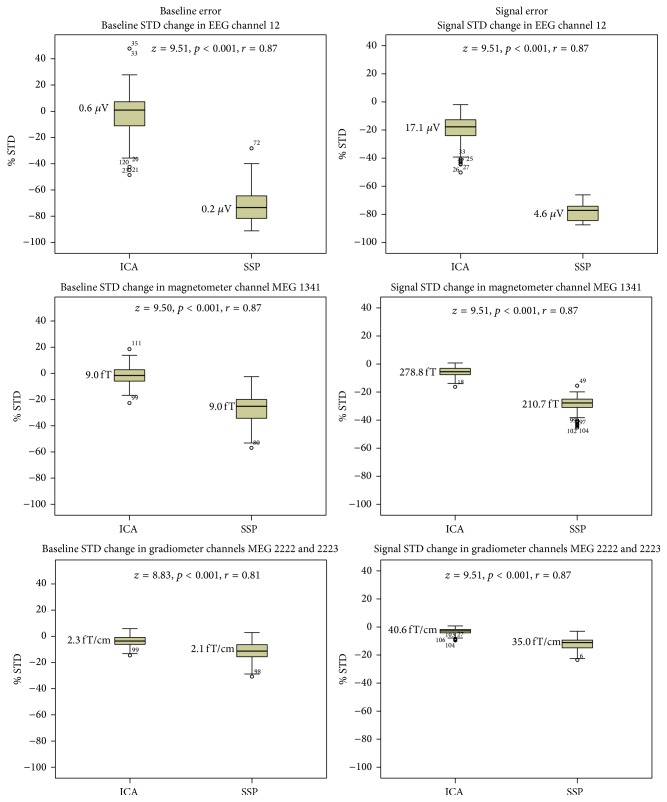
Percentagewise baseline standard deviation (STD) and signal STD reduction achieved by applying ICA or SSP methods in comparison to the level (at 0% STD) in the raw data recording with tSSS only. Median values are shown in microvolts (*μ*V), femtoteslas (fT), and femtoteslas per centimeter (fT/cm). Outliers more than 1.5 interquartile ranges from the edge of the 50% of the cases are denoted with circles. Case numbers are provided for each outlier.

**Figure 5:**
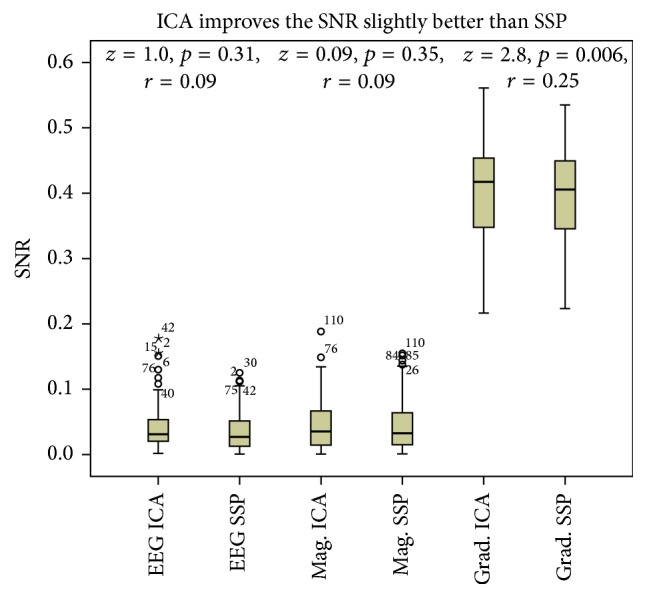
Signal-to-noise ratios (SNR) achieved by applying artifact correction based on Independent Component Analysis (ICA) or Signal Space Projection (SSP). Tukey box plot showing the SNR levels achieved by applying the ICA or SSP method in the signal amplitude peak channel of the EEG, magnetometers (Mag.), and gradiometers (Grad.) (the SNR shows the relationship between the signal and noise level in single trials). Outliers more than 1.5 (circles) or 3.0 (stars) interquartile ranges from the edge of the 50% of the cases are denoted with circles or stars, and case numbers are provided for each outlier.

The SSP method results in lower baseline standard deviation (STD) and signal STD in comparison to the ICA method (see Figure 4). The baseline STD even appears to increase when applying the ICA-based artifact correction, in particular with respect to the EEG and magnetometer channels. However, for the gradiometers, ICA yields slightly but statistically significantly better SNR than that achieved by applying the SSP method (see Figure 5).

The SNR achieved by applying the ICA-based artifact correction is similar to that achieved by applying the SSP method with respect to the EEG and magnetometer channels. However, for the gradiometers applying the ICA method results in statistically significantly and slightly better SNR than that achieved by applying the SSP method (see Figure 5).

## 4. Discussion

We compared the noise suppression results achieved with SSS and tSSS on healthy subjects not wearing magnetized material. MaxFilter with tSSS resulted in better suppression of artifacts from external and nearby noise sources in comparison to SSS. In particular, the application of tSSS instead of SSS was important with respect to the MEG gradiometers, since SSS correction in 6% of the cases resulted in an increase of the noise level in the MEG gradiometer data, and thus the reliability of the gradiometer data decreased in comparison to that before SSS. We also compared the performance of ICA and SSP in reducing internal electrophysiological artifacts, originating from eye movements and heart beats of the participants. The ICA-based artifact correction performed better than the SSP method. The SSP method reduced part of the signal of interest along with the artifacts, and the SNR was slightly higher after applying the ICA method than after applying the SSP method. However, after ICA-based artifact correction the baseline noise level increased, in particular in the EEG and magnetometer channels, which have relatively low SNR in the original data. These findings support both the importance of reducing the bias on measures of evoked-responses with EEG and MEG caused by artifacts and the importance of minimizing the bias introduced by errors in artifact correction methods.

With regard to the suppression of external noise, we here observed that the averaged MMN evoked-response in 6% of the cases with the MEG gradiometers even became more unreliable after applying SSS correction than before. Possibly, the influence of nearby artifacts on the evoked-response can become stronger after the external artifacts have been reduced with the SSS. When tSSS is applied the influence of such nearby artifacts on the evoked-responses would be reduced. In general, it seems relevant to further investigate whether correction methods for reducing one type of artifact, such as SSS, in some cases might enhance the influence of other artifacts on the averaged event-related waveform.

The comparisons of the ICA and SSP methods for suppression of internal artifacts revealed particular biases appearing after the corrections. For the SSP method, there is a risk that the artifacts and signals of interest are not described by orthogonal components [48]. We observed this issue after applying the SSP artifact correction method, and we found that part of the signal of interest was reduced along with the influence of the artifacts. For the ICA method, there is another risk that after correction on channels with relatively low SNR is applied—such as correction on EEG channels, magnetometer channels, and channels located distantly from the signal peaks—additional noise is added to these channels. This happens because the errors in estimating the mixing matrix for the ICA will increase when the SNR decreases [60]. Our results emphasized this issue in showing that the baseline noise level increased after applying the ICA-based correction, in particular in the EEG channels and in the MEG magnetometer channels, and also the difference in SNR between applying ICA and SSP was smaller for the EEG channels and MEG magnetometer channels than for the MEG gradiometer channels, suggesting a relatively smaller improvement for the EEG and MEG magnetometer channels after applying the ICA-based artifact correction.

In summary, our test results suggest that tSSS is recommendable for reducing the influence of artifacts originating from external and nearby sources instead of SSS only. We find that the noise level decreases more with tSSS than with SSS in this sample of EEG and MEG data from healthy participants despite the fact that they were not wearing strongly magnetized materials. For the reduction of internal physiological artifacts, we showed that the highest signal-to-noise ratio (SNR) is achieved with ICA-based artifact correction on the tested sample. However, both ICA- and SSP-based artifact corrections are subject to certain limitations. In particular, one must be aware of the risk when processing data with relatively low SNR, such as EEG and MEG magnetometer data, that artifact correction based on ICA may decrease the interference from artifacts while simultaneously increasing the noise level, due to increasing errors in estimating the mixing matrix in the context of data with lower SNR levels.

## Figures and Tables

**Table 1:** Deviant tones. *Type of deviant* is the type of change applied to the tone, and *description* explains the change. *Occurrence* describes whether the change is presented in different patterns randomly (single) or is present constantly in each following pattern from the first presentation until the next deviant of the same type occurs (consistent). *Tone number* denotes at which tone of the melody pattern the change may occur. *Melodies with deviants* column shows the percentage of melodies containing the particular type of deviant.

Type of deviant	Description	Occurrence	Tone number	Melodies with deviants
Mistuning	3% pitch frequency increase	Single	1st, 2nd, 4th	14%
Timbre	Flute sound	Single	1st, 3rd, 4th, ending	8%
Timing delay	100 ms silent gap before the tone	Single	1st, 2nd, 3rd, ending	8%
Melody modulation	Tone replacement	Consistent	3rd, 4th	12%
Rhythm modulation	Duration switch between two successive tones	Consistent	2nd, 3rd	7%
Transposition	Semitone pitch change of a pattern	Consistent	Initial triad	16%
